# Targeted Metabolomics Analysis on Obstructive Sleep Apnea Patients after Multilevel Sleep Surgery

**DOI:** 10.3390/metabo10090358

**Published:** 2020-09-01

**Authors:** Abdulmohsen Alterki, Shibu Joseph, Thangavel Alphonse Thanaraj, Irina Al-Khairi, Preethi Cherian, Arshad Channanath, Devarajan Sriraman, Mahmoud A. K. Ebrahim, Alaaeldin Ibrahim, Ali Tiss, Fahd Al-Mulla, Anas M. Abdel Rahman, Jehad Abubaker, Mohamed Abu-Farha

**Affiliations:** 1 Department of Otolaryngology Head & Neck Surgery, Zain and Al Sabah Hospitals and Dasman Diabetes Institute, Dasman 15462, Kuwait; abdulmohsen.alterki@dasmaninstitute.org (A.A.); Drmahebrahim91@gmail.com (M.A.K.E.); Aladin289@yahoo.com (A.I.); 2 Special Service Facility Department, Dasman Diabetes Institute, Dasman 15462, Kuwait; shibu.joseph@dasmaninstitute.org (S.J.); sriraman.devarajan@dasmaninstitute.org (D.S.); fahd.almulla@dasmaninstitute.org (F.A.-M.); 3 Department of Genetics and Bioinformatics, Dasman Diabetes Institute, Dasman 15462, Kuwait; alphonse.thangavel@dasmaninstitute.org (T.A.T.); arshad.channanath@dasmaninstitute.org (A.C.); 4 Department of Biochemistry and Molecular Biology, Dasman Diabetes Institute, Dasman 15462, Kuwait; irina.alkhairi@dasmaninstitute.org (I.A.-K.); preethi.cherian@dasmaninstitute.org (P.C.); ali.tiss@dasmaninstitute.org (A.T.); 5 Department of Genetics, King Faisal Specialist Hospital and Research Centre (KFSHRC), Zahrawi Street, Al Maather, Riyadh 11211, Saudi Arabia; aabdelrahman46@kfshrc.edu.sa; 6 Department of Biochemistry and Molecular Medicine, College of Medicine, Al Faisal University, Riyadh 11533, Saudi Arabia; 7 Department of Chemistry, Memorial University of Newfoundland, St. John’s, NL A1B 3X7, Canada

**Keywords:** obstructive sleep apnea, metabolomics, triglycerides, phosphocholines, ceramides, apnea hypopnea index, polysomnography, lipid metabolism, multilevel sleep surgery

## Abstract

Background: Obstructive sleep apnea (OSA) is caused by partial or complete obstruction of the upper airways. Corrective surgeries aim at removing obstructions in the nasopharynx, oropharynx, and hypopharynx. OSA is associated with an increased risk of various metabolic diseases. Our objective was to evaluate the effect of surgery on the plasma metabolome. Methods: This study included 39 OSA patients who underwent Multilevel Sleep Surgery (MLS). Clinical and anthropometric measures were taken at baseline and five months after surgery. Results: The mean Apnea-Hypopnea Index (AHI) significantly dropped from 22.0 ± 18.5 events/hour to 8.97 ± 9.57 events/hour (*p*-Value < 0.001). Epworth’s sleepiness Score (ESS) dropped from 12.8 ± 6.23 to 2.95 ± 2.40 (*p*-Value < 0.001), indicating the success of the surgery in treating OSA. Plasma levels of metabolites, phosphocholines (PC) PC.41.5, PC.42.3, ceremide (Cer) Cer.44.0, and triglyceride (TG) TG.53.6, TG.55.6 and TG.56.8 were decreased (*p*-Value < 0.05), whereas lysophosphatidylcholines (LPC) 20.0 and PC.39.3 were increased (*p*-Value < 0.05) after surgery. Conclusion: This study highlights the success of MLS in treating OSA. Treatment of OSA resulted in an improvement of the metabolic status that was characterized by decreased TG, PCs, and Cer metabolites after surgery, indicating that the success of the surgery positively impacted the metabolic status of these patients.

## 1. Introduction

The rise in worldwide obesity rates has also been paralleled by an increase in obstructive sleep apnea (OSA) [1]. OSA is associated with disturbed sleep and intermittent hypoxia resulting from partial or complete cessation of breathing during sleep. OSA causes daytime lethargy and has been linked to road traffic accidents [2], as well as reduced productivity at the workplace [3]. OSA rates, reported in the literature, are extremely variable, mainly due to differences in methods of assessment. The gold standard for the diagnosis of OSA is the polysomnography (PSG) test, which estimates the Apnea-Hypopnea Index (AHI) [1]. AHI is a composite index that is made up of the apnea index (AI), which is defined as a complete cessation for ≥10 s, as well as the hypopnea index (HI), which is defined as a reduction in the respiratory effort with a ≥4% oxygen desaturation. OSA can also be estimated using the Epworth Sleeping Scale (ESS), which is a subjective self-administered questionnaire that estimates the daytime sleepiness by responding to the likelihood (0–3 points) of falling asleep while involved in eight different daily activities. Multiple treatment modalities exist for OSA, including weight loss for overweight people, continuous positive airway pressure (CPAP), oral devices, as well as surgeries such as bariatric and upper airway surgeries [4,5,6]. OSA usually involves one or more upper airway levels. Thus, multilevel sleep surgery (MLS) in a single-stage procedure has been developed as a surgical treatment method for OSA patients that require surgery. The success rate for the procedure is 60% [7,8].

Additionally, soaring rates of OSA are disconcerting as they are associated with an increased risk of multiple chronic diseases, including metabolic syndrome, Type 2 Diabetes (T2D) and cardiovascular diseases (CVD) [9,10,11,12]. It has been linked to the dysregulation of multiple metabolic-related pathways such as inflammation, oxidative stress, and insulin resistance [13,14,15,16,17,18,19]. OSA has also been demonstrated to dysregulate the triglyceride metabolism, which plays a pivotal role in linking inflammation, oxidative stress, and insulin resistance [20]. Hypoxia is one of the hallmarks of OSA. Hypoxia is marked by the downregulation of the activity of lipoprotein lipase (LPL), thereby regulating the hydrolysis of triglyceride-rich lipoprotein into fatty acids [21,22,23]. LPL activity is regulated by various factors, including angiopoietin-like (ANGPTL) proteins, such as ANGPTL4, and 8, which were increased in people with OSA [24,25,26,27]. Other classes of metabolites have been demonstrated to be affected by OSA. For example, acylcarnitines, glycerophospholipids, and sphingomyelin were found to be increased in the urine of moderate and severe OSA patients when compared to controls [28]. In an earlier study, Ferrarini et al. utilized metabolomics to quantify various phospholipids in people diagnosed with severe and non-severe OSA [29]. Metabolomics is an emerging technique that has been fundamental in enhancing our understanding of global changes in metabolic pathways by allowing the quantification of various metabolites in clinical and personalized medicine [30]. Metabolomics is mainly focused on the quantification and identification of low molecular weight metabolites that can be used for disease diagnosis, drug targets, as well as a better understanding of the cellular pathways involved in disease pathophysiology [31,32,33]. In order to better understand the role of various classes of metabolites in OSA, we studied the metabolic changes using a targeted panel used commercially on OSA before and after MLS.

## 2. Results

### 2.1. Study Population Characteristics

The study population was composed of 39 patients who underwent MLS. The population characteristics are shown in Table 1. The average time of the repeated investigations was five months after the surgery. Overall, there were no significant changes in the Body Mass Index (BMI) and blood pressure. No significant changes were observed in total cholesterol, High Density Lipprotein (HDL), Low Density Lipoprotein (LDL), Fasting Glucose (FG) or Glycosylated Hemoglobin A1C (HbA1c). A slight reduction in the Triglyceride (TG) level was observed, though it was not significant.

### 2.2. Polysomnographic Data

**Figure 1:**
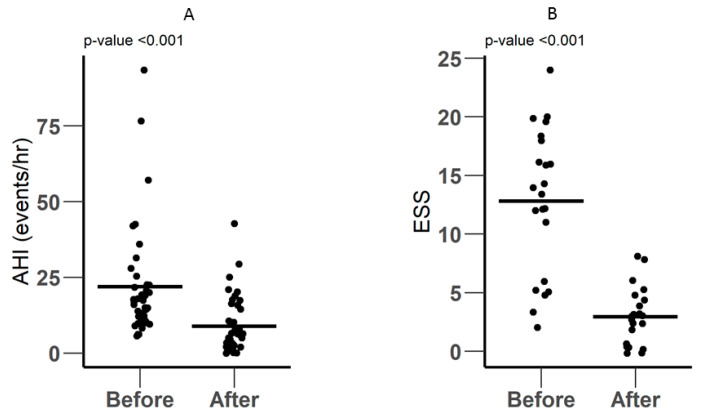
Polysomnographic data showing (**A**) the AHI score and (**B**) the ESS score, before and after the surgery. OSA was diagnosed based on an AHI >5 events/hour.

The AHI score was used for the diagnosis of OSA. On average, the AHI and ESS scores were significantly lower after surgery. Upon defining a threshold of AHI ≥30 to define patients as having severe sleep apnea, it was found that only seven patients could be categorized into the severe group, with 32 in the non-severe group. The mean AHI score of the non-severe group was 14.97 ± 5.79, and that of the severe group was 54.20 ± 23.03 (Figure 1A). The AHI dropped from 22.0 ± 18.5 events/hour to 8.97 ± 9.57 events/hour (*p*-Value <0.001), while the ESS score dropped from 12.8 ± 6.23 to 2.95 ± 2.40 (*p*-Value < 0.001) (Figure 1B).

### 2.3. Metabolomics Analysis of People with OSA after MLS

The Biocrates kit p400 was used to quantify a total of 408 plasma metabolites. Out of these metabolites, a total number of 256 were identified from all classes of metabolites. The quantified metabolites are shown in Supplementary Table S1. The quantified metabolites included amino acids, biogenic amines, acylcarnitines, glycerophospholipids phosphatidylcholines and lysophosphatidylcholines, glycerides (triglycerides and diglycerides), hexoses (including glucose), cholesterol esters, and glycerides (triglycerides and diglycerides).

### 2.4. Metabolomics Global Analysis

**Figure 2:**
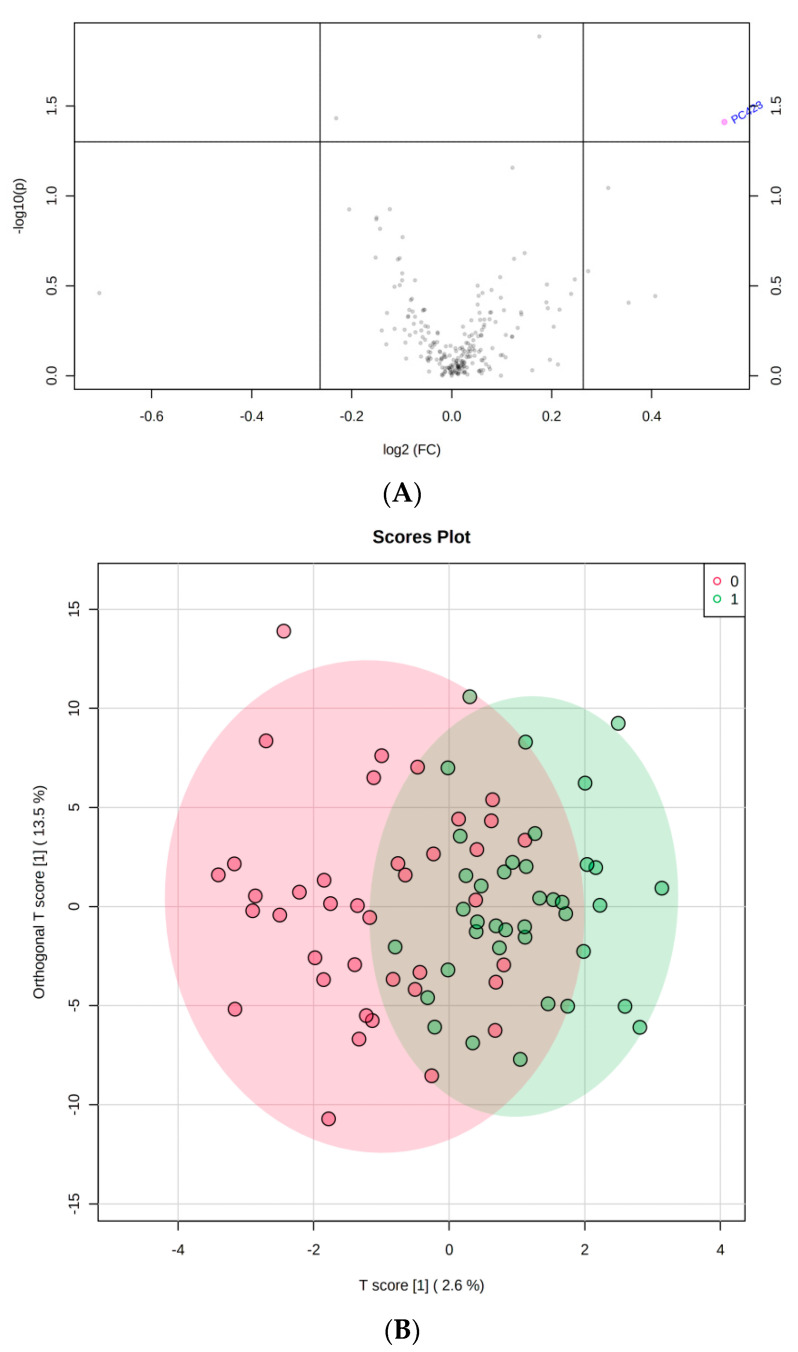
(**A**) volcano plot and (**B**) OPLSDA.

Using this analysis, a total of 256 metabolites were detected in the positive ionization mode in both study groups and underwent a univariant analysis. After normalizing the samples to the mean of the signal, a log-transformation, and Parito scaling, the population of the samples and analytes was normally distributed (Supplementary Figure S1) A volcano plot showed only one significant metabolite PC482 based on a fold change (x-axis) and FDRp-value (y-axis) cutoff of 1.2 and 0.05, respectively (Figure 2A). This profile was not strong enough to distinguish between both study groups using orthogonal PLSDA, as shown in Figure 2B.

### 2.5. Differentially Expressed Triglyceride Metabolites

**Figure 3:**
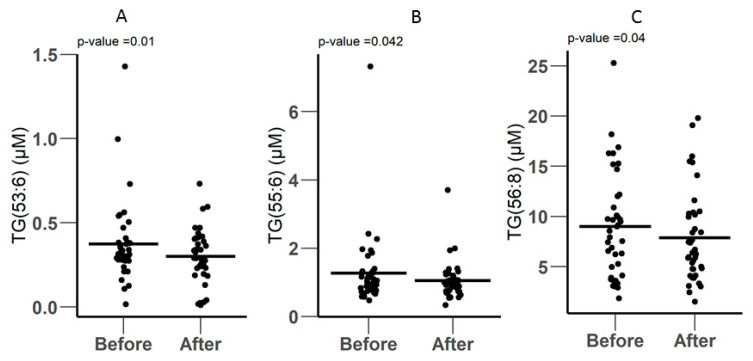
(**A**–**C**) Differentially expressed TGs before and after surgery, as measured by LC-MS using the biocrates P400 kit.

The Biocrates P400 kit includes 60 glycerides (42 triglycerides and 18 diglycerides). A total of 51 metabolites were quantified, as shown in Supplementary Table S1, including 36 triglycerides and 15 diglycerides. Three TG metabolites were significantly decreased after surgery: TG.53.6, TG.55.6, and TG.56.8. TG.53.6 was reduced from 0.375 ± 0.242 µM to 0.302 ± 0.162 µM (*p*-Value = 0.01) after surgery (Figure 3A). TG.55.6 was also decreased from 1.27 ± 1.10 µM to 1.05 ± 0.554 µM after surgery (*p*-Value = 0.042) (Figure 3B). The third TG metabolite was TG.56.8, which was decreased from 9.00 ± 5.24 to 7.89 ± 4.57 after surgery (*p*-Value = 0.04), as shown in (Figure 3C).

### 2.6. Differentially Expressed Phospholipid Metabolites

**Figure 4:**
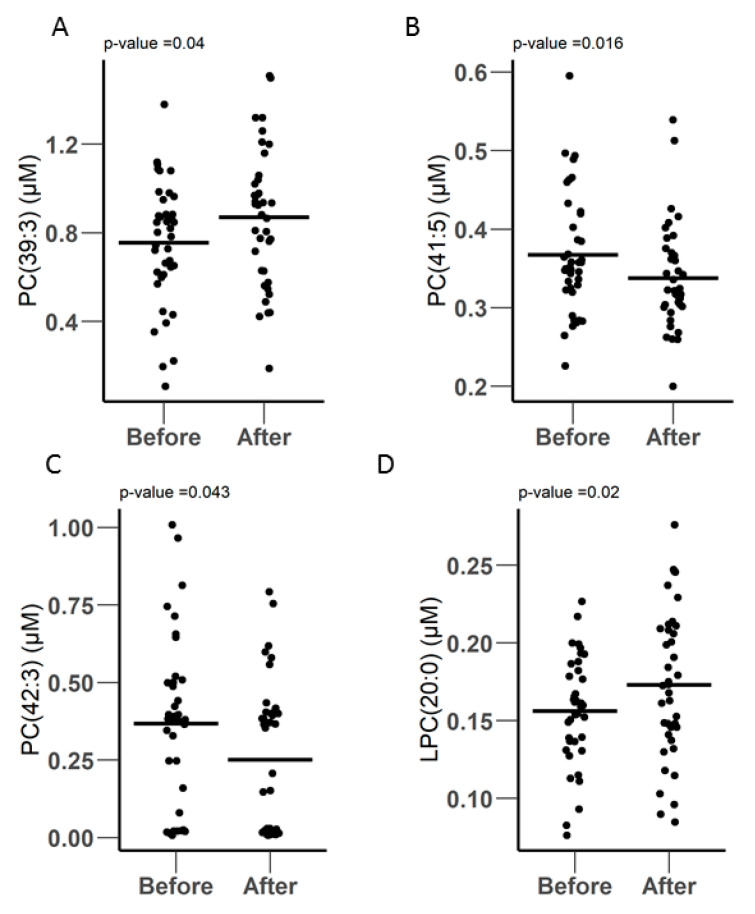
(**A**–**C**) Differentially expressed PCs and (**D**) LPC before and after surgery, as measured by LC-MS using the biocrates P400 kit.

Most of the metabolites included in the Biocrates P400 kit were glycerophospholipids, including 172 phosphatidylcholines and 24 lysophosphatidylcholines. A total of 123 metabolites were quantified, including 102 PCs and 21 LPCs. Three PCs were differentially expressed, including PC.39.3, which was significantly increased in people from 0.756 ± 0.277 µM to 0.871 ± 0.307 µM after surgery (*p*-Value = 0.04) (Figure 4A). The other two PCs decreased after surgery: PC.41.5, which was reduced from 0.367 ± 0.0769 µM to 0.338 ± 0.0657 µM after surgery (*p*-Value = 0.016) and PC.42.3, which was reduced from 0.368 ± 0.269 µM to 0.251 ± 0.242 µM (*p*-Value = 0.043) after surgery (Figure 4B,C, respectively). The only LPC metabolite that was differently expressed was LPC.20.0, which increased after surgery from 0.156 ± 0.0347 µM to 0.173 ± 0.0475 µM (*p*-Value = 0.02), as shown in Figure 4C.

### 2.7. Differentially Expressed Sphingolipid Metabolites

**Figure 5:**
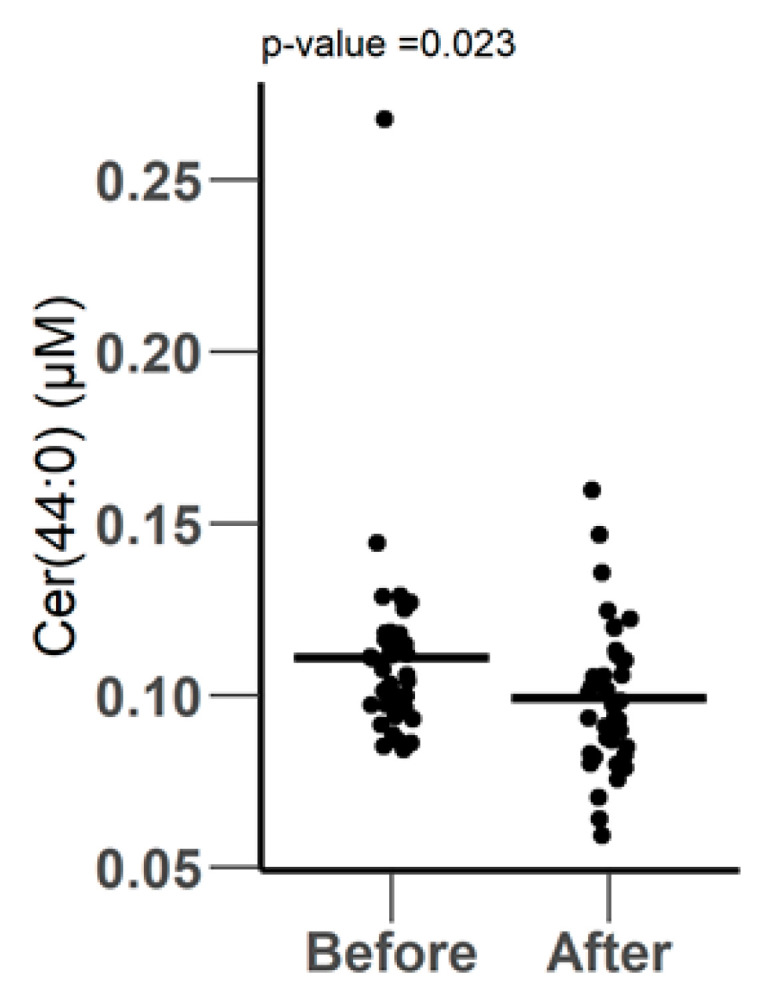
Differentially expressed Ceramides before and after surgery, as measured by LC-MS using the biocrates P400 kit.

A total of 55 acylcarnitines were included in the Biocrates P400 kit. Out of them, 48 acylcarnitines were quantified. The only significantly expressed metabolite was Cer.44.0, which was reduced from 0.11 ± 0.03 µM to 0.09 ± 0.02 µM after surgery (*p*-value = 0.023), as shown in Figure 5.

### 2.8. Biomarker Evaluation

**Figure 6:**
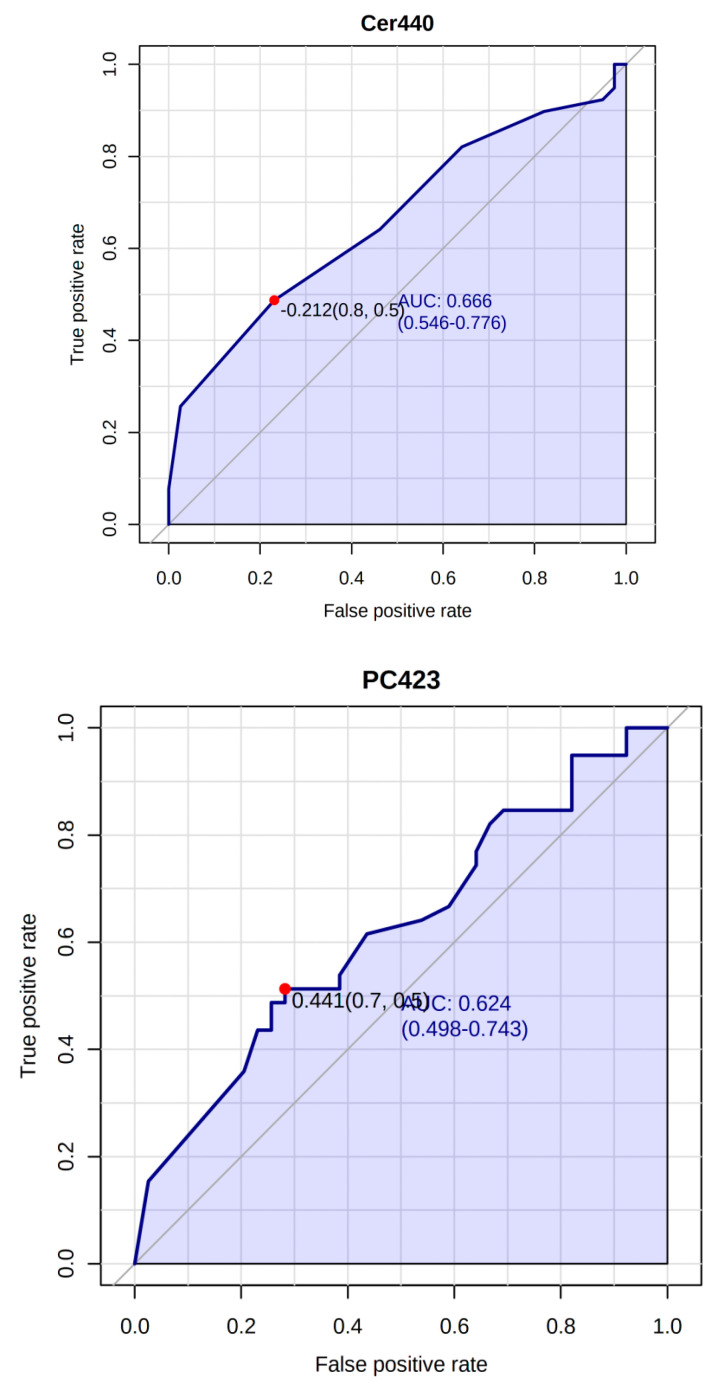
The area-under-the-curve (AUC) of Receiver Operating Characteristic (ROC) analysis showing Cer440 and PC423 specificity and selectivity to distinguish between the study groups.

The biomarker and the ratio between the top 20 candidates were selected using the area-under-the-curve (AUC) of the Receiver Operating Characteristic (ROC) analysis, with a sample classification based on PLSDA with a latent number 2 [34,35]. The sample-set was first divided into a test set containing 70% of injections, and a validation set with the remaining 30%. The test set was employed for the selection of biomarker candidates and the construction of the classification model, whereas the validation test was retained for an independent evaluation of the performance of the model. The ROC analysis showed a Cer440 and PC423 specificity and selectivity for distinguishing between the study groups, with AUC 0.666 and 0.624, respectively (Figure 6). The ratio between the Cer440/CE172 and PC423/PC-O383 showed a slight increase in ACU with 0.681 and 0.683, respectively (Supplementary Figure S1).

## 3. Discussion

Increasingly, OSA is becoming a significant health problem that is aggravated by the increasing obesity rates worldwide. Its proper diagnosis requires the use of an expensive and lengthy procedure known as the PSG test. Metabolites are showing great promise in advancing our understanding of the pathophysiology as well as diagnostic biomarkers.

In this study, we analysed patients who were treated for OSA through MLS by using a targeted metabolomics panel on blood serum, in order to explore potential biomarkers for surgical efficiency. Initially, a global statistical analysis between the study groups showed the changes caused by the operation. Based on the explored metabolic panel, there were no dramatic changes between the study groups. A potential biomarker was filtered out based on a univariate analysis. The combination between multiple metabolites revealed a stronger selectivity and sensitivity biomarker, based on the ROC curve analysis. The surgical procedures that the participants received were a different combination of surgeries, as indicated by their level of upper airway obstruction, which included tonsillectomy, adenoidectomy, and septoplasty (the complete list of surgeries is in the method section). There was a significant reduction in the AHI values following surgery. We compared the metabolome profile of people with a successful surgical outcome before and after surgery. Most metabolites did not show any significant change after surgery. Nonetheless, the majority of the differentially expressed metabolites showed a reduction in their level after surgery. These metabolites were triglycerides, sphingolipids, as well as phosphocholines. It is important to note that the BMI of the people under study did not change before and after surgery, thus excluding the impact of obesity in these findings.

Several epidemiological studies have emphasised an increased risk of T2D in people diagnosed with OSA, independent of obesity [9,10,16,17]. It is known that hypoxia leads to increased inflammation, as well as increased insulin resistance. It also mediates the activation of the hypothalamic-adrenal axis and reduces β-cell function [36]. This is indicated in the high prevalence of OSA amongst people with T2D, which has been reported to be at minimum 24%, and which can reach about 86% [37]. This is particularly alarming as OSA has been associated with increased vascular complications, as well as worse glycaemic control [38,39,40,41]. On the other hand, T2D has also been identified as a risk factor for the development of OSA, suggesting a bidirectional relationship between T2D and OSA. In a retrospective study examining 360,250 people with T2D and 1,296,489 people without T2D, Subramanian et al. showed that T2D patients are at an increased risk of OSA, especially male patients with a high BMI with diabetic foot diseases, depression, hypertension, or CVD, as well as patients taking insulin [37].

OSA is an overly concerning and underdiagnosed disease, especially in a population with high rates of obesity and diabetes. For example, in the Arabian Gulf region, obesity and overweight rates can reach up to 90% in countries like Kuwait, and T2D is around 20% [42,43]. As a result, an improved understanding of OSA-associated risk with T2D and other chronic diseases is critical because OSA can be treated with various procedures. Multiple treatment modalities exist for OSA, including weight loss in overweight people, CPAP, oral devices, as well as surgeries, such as bariatric and upper airway surgeries (such as MLS) [4,5,6]. The most effective surgical procedure to reduce AHI in MLS is tonsillectomy. Studies have shown that MLS with tonsillectomy was effective in reducing the AHI in 58% of patients, while MLS performed without tonsillectomy was only effective in reducing the AHI in 19% of patients [7,8]. All our patients underwent tonsillectomy, combined with other surgical procedures. It is important to highlight that our OSA patients included in this study had a wide range of AHI values, which constitutes one of the limitations of this study. However, it is important to highlight the fact that MLS surgery has resulted in a dramatic reduction in AHI that allows us to examine the difference in metabolites before and after surgery. Given the fact that tonsillectomy is a very common surgical procedure, particularly in children, its utility in treating OSA (as it improves the airflow and improves breathing) is also combined with an improved metabolic status [44].

The metabolomics analysis showed a reduction in multiple triglyceride species after surgery. OSA has been linked to the triglyceride metabolism, particularly through intermittent hypoxia (IH), which is one of the hallmarks of OSA [45,46]. In people with OSA, the repeated apnea and hypopnea events, which result in a complete or partial cessation of breathing due to the collapse of the upper airway, induce hypoxia [45,46]. The duration of such events determines the reduction in oxygen saturation and the severity of the diurnal consequences of OSA. IH has been linked to dysregulated triglyceride metabolism through the inhibition of LPL in adipose tissue [1,14,15,47]. It was also postulated that the inhibition of adipose tissue LPL, rather than elevated hepatic TG secretion, was responsible for the dysregulated TG metabolism under hypoxic conditions [22]. LPL is responsible for the hydrolysis of TG from TG-rich chylomicrons and VLDL to generate energy [48,49]. We have recently shown that two of the important regulators of LPL activity, ANGPTL4 and 8, were increased in people with OSA [24]. Others have also shown that ANGPTL4 was increased in people with OSA [21]. ANGPTL3, 4, and 8 are inhibitors of LPL activity [48,50]. ANGPTL4 is increased under hypoxia through the master regulator of the hypoxic response, hypoxia-inducible factor 1 alpha (HIF-1α) [51,52,53]. Drager et al. showed that the IH-driven increase in ANGPTL4 expression led to atherosclerosis in an apolipoprotein E (apoE) knockout mouse model [54].

Furthermore, the same group has recently shown that people with severe OSA exhibited delayed lipoprotein remnants removal, as well as decreased lipolysis of TG-rich particles. Both processes were positively correlated with the severity of IH and were enhanced by CPAP treatment [55]. Our data also point in the same direction and highlight the beneficial impact of MLS in the treatment of OSA. The main advantage of this procedure is the permanent correction to the OSA problem in people undergoing the surgery. The current study is the first report to shed light on the impact of MLS on the metabolic profile of people before and after the surgery, highlighting the impact of this surgery on TG and other metabolites.

In our current study, the second class of metabolites that were shown to be reduced were the phospholipids. Phospholipids, also called glycerophospholipids, are important structural components in the lipid bilayer of the plasma membrane. Phosphocholines are a class of phospholipids that are also part of the plasma membrane and play an important role as signaling molecules [56,57,58]. Interestingly, two PCs were increased following the surgical treatment of OSA. Previously, Lebkuchen et al. showed that people with OSA had a reduction in PCs [59]. They linked the observed reduction in PCs to the increased damaging activity of various phospholipase A1 (PLA1), A2 (PLA2), and C (PLC), which are activated under hypoxic conditions. PLA2 is required for the remodeling and repair of cell membranes. The activation of PLA2 in children with OSA was connected to endothelial dysfunction [60].

Finally, in our study, one species of Lysophosphatidylcholine (LPC) was increased in OSA people after surgery. LPCs are related to PCs as they are derived from PCs’ turnover in the circulation by PLA2. Generally, LPCs have been positively associated with cardiovascular and neurodegenerative diseases. Species of this family have been recognized as diagnostic markers for myocardial infarction (LPC 17:0 and LPC 18:2) and have been suggested to be associated with systemic inflammation [61]. They were also linked to promoting fatty acid-induced insulin resistance. In line with our data, Lebkuchen et al. showed that species of LPC were upregulated by OSA [59]. This finding could possibly show a different pattern of expression of LPCs or could be due to one of the main limitations of this study, which is the limited number of participants dictated by the nature of our study, which involved surgical intervention.

In conclusion, the current study demonstrated the positive impact of MLS on the treatment of OSA, where AHI values were dramatically reduced after surgery. It also exhibited a reduction in TG metabolites that could be indicative of an improved metabolic state after OSA treatment. As this targeted panel does not show the exact change in these patients, an untargeted approach will be conducted to cover more metabolites and pathways that are involved in oxidative stress.

## 4. Materials and Methods

### 4.1. Study Population and Ethical Statement

The study was approved by the ethical review board of the Dasman Diabetes Institute and conducted in accordance with the Declaration of Helsinki ethical guideline Study number RA 2015-043. Written informed consent was obtained from all subjects prior to participation in the study. All patients who underwent MLS were followed-up for at least six months. Inclusion criteria were: those who underwent MLS and completed a pre-operative and post-operative level 1 polysomnography (PSG), pre-operative and post-operative ESS, pre-operative and post-operative blood metabolites, and we recorded their medical history and patient’s data, such as Body Mass Index (BMI). Exclusion criteria were patients with medical diseases, such as diabetes, hypertension, and cardiovascular disorders. The total number of participants who met our inclusion criteria was 39.

### 4.2. OSA Assessment and the Surgery Procedures

The participants were diagnosed with OSA according to a level 1 polysomnography (PSG) if their Apnea-Hypopnea Index (AHI) was more than 5 events/h. Furthermore, we evaluated other parameters of the PSG, such as apnea events and hypopnea events. The PSG sleep was performed at baseline and at least three months post-operatively. Moreover, the participants completed the ESS during the time of their PSG sleep study. The BMI was calculated using the standard BMI formula: body weight (kg)/height (m^2^). All participants who were undergoing surgery were carefully selected, and an individualized procedure was performed according to the site of the obstruction (oropharynx, hypopharynx, and/or nasopharynx). The principal author (ALT) was the sole sleep apnea surgeon for all the patients. After the individualized MLS was planned for the patient, the procedure was started with Drug-Induced Sleep Endoscopy (DISE) in order to further identify the obstruction sites. Afterward, we proceeded with MLS, aiming to relieve the site of obstruction that was present in the patients (oropharynx, hypopharynx, and/or nasopharynx). Upon completion of the surgical procedures, the patients were kept in the hospital for further evaluation and monitoring and were discharged once they were stable. They were followed in the sole sleep surgeon outpatient clinic for two weeks, six weeks, three months, and six months post-operatively. If any further follow-up visits were needed, the patients scheduled their appointments. They repeated the level 1 PSG, ESS, and blood investigations at least three months after the surgical procedure.

### 4.3. Blood Collection and Anthropometric and Biochemical Measurements

A fasting blood sample was collected twice from each participant, before the MLS operation and five months after the operation. Blood samples were collected in a vacutainer EDTA tube where the plasma was separated by centrifugation at 400× *g* for 10 min. The plasma was then aliquoted and stored at −80 °C until it was assayed as previously reported [50,62,63]. Blood pressure was measured using an Omron HEM-907XL digital sphygmomanometer. The mean blood pressure of the three readings was recorded. Clinical parameters including the fasting blood glucose (FBG), triglyceride (TG), total cholesterol, low-density lipoprotein (LDL), and high-density lipoprotein (HDL) were measured using a Siemens Dimension RXL chemistry analyzer (Diamond Diagnostics, Holliston, MA, USA). The glycated hemoglobin, HbA1c, was measured using a Variant^TM^ device (Bio-Rad, Hercules, CA, USA).

### 4.4. Metabolomics Analyses

Plasma metabolites were analyzed by a quantitative targeted metabolic analysis method, using the Absolute IDQ-p400 HR kit (Biocrates Life Science AG, Innsbruck, Austria). This kit provides quantitative data on 408 metabolites that span 11 classes of metabolites, including 21 amino acids, 21 biogenic amines, 55 acylcarnitines, 196 glycerophospholipids (172 phosphatidylcholines and 24 lysophosphatidylcholines), 60 glycerides (42 triglycerides and 18 diglycerides), hexoses (including glucose), and 14 cholesterol esters. Quantification is based on a combination of Liquid Chromatography-High Resolution Mass Spectrometry (LC-HRMS) that is used for the quantitation of amino acids and biogenic amines. The second part is used to quantify all other metabolites using a Flow Injection Analysis-HRMS (FIA-HRMS). The quantification of the LC-HRMS metabolites is based on a 7-point calibration curve, unlike the FIA-HRMS, which uses a single point calibrator. Quality control samples were included in each plate, which included injecting a zero sample, three blanks, and three quality control (QC) levels (QC 1–3), of which the QC2 medium level was injected five times.

According to the manufacturing instructions, the calibrators, quality controls, and internal standard were diluted to the required concentration. After that, all samples, including the plasma samples and QCs, were centrifuged at 2750× *g* at 4 °C. Briefly, 10 μL of plasma was pipetted into a 96-well kit plate containing the internal standards, and the samples were dried for 30 min using a Nitrogen Evaporator. The samples were then derivatized with 50 μL of 5% PITC solution in water:ethanol:pyridine at a ratio of 1:1:1. The plate was incubated for 20 min before drying again under nitrogen for 60 min. After that, the samples were extracted by addition of 300 ul of 5 mM ammonium acetate in methanol and shaking at 450 rpm for 30 min at room temperature and were collected in a capture plate for a FIA-HRMS run, after which 250 μL of FIA mobile phase was added to each well of the original capture plate. A total of 150 μL from the capture plate was then transferred to another plate and diluted with 150 μL LC-MS grade water for the LC-HRMS run. The samples were then analyzed on a high-resolution Q-Exactive HF hybrid quadrupole-Orbitrap mass spectrometer (Thermo Scientific), which was equipped with an electrospray ionization source coupled to a Vanquish Duo UHPLC system. The instruments were controlled using XCalibur 4.1 and Q-Exactive HF tune software V 2.9 SP4All. All parameters were set according to the Biocrates instructions. All solvents used were LC-MS hyper grade from Thermo Fisher Scientific. The analysis was done in the positive and negative ionization modes for both LC-HRMS and FIA-HRMS, respectively. The mobile phase A was 0.2% formic acid in H_2_O, and the mobile phase B was 0.2% formic acid in acetonitrile. The LC-HRMS chromatographic program was 5.8 min gradient at 0.8 mL/min flowrate at 50 °C. In the FIA-HRMS run, 20 μL of the sample was injected and analyzed for 3 min at 0.05 mL/min for the first 1.6 min, then increased to 0.2 mL/ min for 1.2 min, and then decreased back to 0.05 mL/min for the rest of the program. The LC-HRMS data was preprocessed via XCalibur Quan 4.1 software. All data from the three runs for each sample was processed using the Biocrates Met*I*DQ Nitrogen software. The statistical analysis was performed with the Met*I*DQ StatPack module.

### 4.5. Statistical Analysis

Based on a criterion of a fold-change greater than 1.5 or less than 0.67 with a false discovery rate adjusted *p*-value of less than 0.05, a univariate analysis (volcano plot) was performed for each binary comparison to identify significantly differentially expressed metabolites. The x-axis, on the volcano plot, represents the fold change (FC) between two comparison groups, and the y-axis represents the *p*-value. Multivariate analyses, including the Orthogonal Projections to Latent Structures Discriminate Analysis (OPLS-DA), were done using MetaboAnalyst.

The global data analysis was performed using MetaboAnalyst version 3.0 (McGill University, Montreal, QC, Canada). The raw data were normalized to the sample total median to ensure all samples were normally distributed, log-transformed, and Pareto-scaled, and the receiver operating characteristic (ROC) curves were constructed using the random forest method.

To assess the normality of the data, the Shapiro Wilk test was performed. Based on the results of the normality test, a paired Student’s t-test or Wilcoxon rank-sum test was used for comparisons between subjects before and after upper airway surgery. All data were reported as mean ± standard deviation. Statistical assessments were two-sided and considered significant at *p* <0.05. All analyses were performed using R: A Language and Environment for Statistical Computing (version 3.6.1).

## Figures and Tables

**Table 1:** Characteristics of all people included in the study before and after surgery.

	Before Surgery (*n* = 39)	After Surgery (*n* = 39)	*p*-Value
**AGE Year**			
**Mean (SD)**	40.0 (10.5)		NA
**Median [Min, Max]**	40.0 [24.0, 65.0]		
**BMI Kg/m^2^**			
**Mean (SD)**	30.2 (4.41)	29.6 (4.61)	0.121
**Median [Min, Max]**	30.0 [20.2, 38.3]	30.0 [19.0, 38.5]	
**SBP**			
**Mean (SD)**	125 (12.7)	124 (13.0)	0.686
**Median [Min, Max]**	125 [97.0, 149]	124 [99.0, 151]	
**DBP**			
**Mean (SD)**	73.1 (10.2)	76.7 (9.79)	0.248
**Median [Min, Max]**	72.0 [55.0, 92.0]	77.5 [57.0, 94.0]	
**AHI Events/Hour**			
**Mean (SD)**	22.0 (18.5)	8.97 (9.57)	<0.001
**Median [Min, Max]**	17.0 [5.70, 93.5]	6.10 [0, 42.8]	
**AI Events/Hour**			
**Mean (SD)**	2.30 (2.62)	3.64 (6.86)	0.862
**Median [Min, Max]**	0.900 [0.100, 7.10]	0.700 [0.100, 15.9]	
**HI Events/Hour**			
**Mean (SD)**	15.8 (10.1)	6.30 (4.93)	0.193
**Median [Min, Max]**	13.5 [4.50, 50.0]	5.80 [0, 15.4]	
**ESS**			
**Mean (SD)**	12.8 (6.23)	2.95 (2.40)	<0.001
**Median [Min, Max]**	13.5 [2.00, 24.0]	3.00 [0, 8.00]	
**TOTAL CHOL mmol/L**			
**Mean (SD)**	4.96 (1.30)	4.96 (1.30)	0.935
**Median [Min, Max]**	5.15 [2.90, 9.20]	4.80 [3.30, 9.90]	
**HDL mmol/L**			
**Mean (SD)**	1.10 (0.238)	1.10 (0.260)	0.848
**Median [Min, Max]**	1.05 [0.770, 1.64]	1.11 [0.320, 1.54]	
**LDL mmol/L**			
**Mean (SD)**	3.19 (1.21)	3.24 (1.37)	0.976
**Median [Min, Max]**	3.30 [1.30, 7.50]	3.10 [1.40, 8.70]	
**TG mmol/L**			
**Mean (SD)**	1.47 (0.945)	1.36 (0.411)	0.99
**Median [Min, Max]**	1.26 [0.470, 5.75]	1.25 [0.650, 2.06]	
**FG mmol/L**			
**Mean (SD)**	5.77 (1.23)	5.77 (1.08)	0.794
**Median [Min, Max]**	5.50 [4.70, 11.4]	5.55 [4.70, 9.70]	
**HbA1c %**			
**Mean (SD)**	5.69 (0.698)	5.59 (0.552)	0.76
**Median [Min, Max]**	5.60 [4.60, 8.40]	5.50 [4.60, 7.70]	

BMI: Body Mass index, SBP: Systolic Blood Pressure, DBP: Diastolic Blood Pressure, AHI: Apnea-Hypopnea Index, AI: Apnea Index, HI: Hypopnea index, ESS: Epworth Sleepiness Scale, TOTAL CHOL: Total Cholesterol, HDL: High-Density Lipoprotein, LDL: Low-Density Lipoprotein, TG: Triglycerides, FG: Fasting Glucose, HbA1C: Glycosylated Hemoglobin A1C.

## Supplementary Materials

The following are available online at https://www.mdpi.com/2218-1989/10/9/358/s1, Figure S1: The area-under-the-curve (AUC) of Receiver Operating Characteristic (ROC) analysis showing ratio between the Cer440/CE172 and PC423/PC-O383 specificity and selectivity to distinguish between the study groups. Table S1: OSA Metabolomics Pre-Post Surg.

## Abbreviations

AI	Apnea Index
HI	Hypoapnea Index
AHI	Apnea/hypopnea index
BMI	Body mass index
CV	Coefficients of variation
HDL	High-density lipoprotein
LDL	Low-density lipoprotein
ELISA	Enzyme-linked immunosorbent assay
TC	Total cholesterol
FBG	Fasting blood glucose
HbA1C	Glycated haemoglobin
ANGPTL	Angiopoietin-like protein
SBP	Systolic blood pressure
DBP	Diastolic blood pressure
TG	Triglyceride
IH	Intermittent hypoxia
MLS	Multilevel Sleep Surgery
PSG	Polysomnography
CPAP	Continuous Positive Airway Pressure
