# Supplementary material for: Targeted Metabolomics Analysis on Obstructive Sleep Apnea Patients after Multilevel Sleep Surgery
Source: Metabolites. 2020 Sep 1;10(9):358. doi: 10.3390/metabo10090358 (PMC7569907; doi:10.3390/metabo10090358)
Supplement: Supplementary file 1 [file metabolites-10-00358-s001.zip › metabolites-882954-supplementary.pdf]

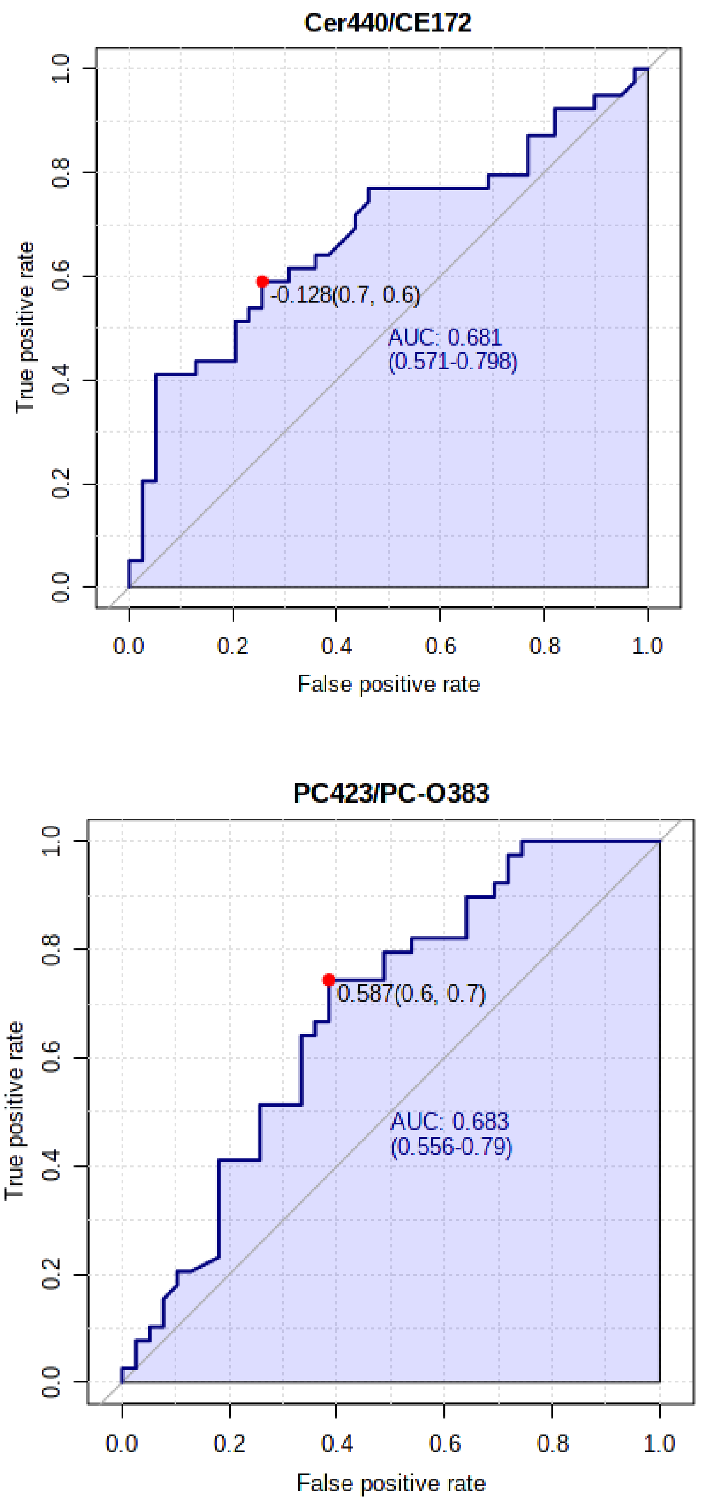

Supplementary Figure 1: The area-under-the-curve (AUC) of Receiver Operating Characteristic (ROC) analysis showing ratio between the Cer440/CE172 and PC423/PC-O383 specificity and selectivity to distinguish between the study groups.
